# Impact of Biochar Amendment on Soil Properties and Organic Matter Composition in Trace Element-Contaminated Soil

**DOI:** 10.3390/ijerph19042140

**Published:** 2022-02-14

**Authors:** José M. De la Rosa, Arturo Santa-Olalla, Paloma Campos, Rafael López-Núñez, José A. González-Pérez, Gonzalo Almendros, Heike E. Knicker, Águeda Sánchez-Martín, Elena Fernández-Boy

**Affiliations:** 1 Instituto de Recursos Naturales y Agrobiología de Sevilla, Consejo Superior de Investigaciones Científicas (IRNAS-CSIC), Reina Mercedes Av. 10., 41012 Seville, Spain; pcampos@irnas.csic.es (P.C.); rlnunez@irnase.csic.es (R.L.-N.); jag@irnase.csic.es (J.A.G.-P.); knicker@irnase.csic.es (H.E.K.); agueda.sanchez@irnas.csic.es (Á.S.-M.); 2 Microal S.L., Castilleja de la Cuesta Av., 5, 41110 Bollullos de la Mitación, Spain; arturosantaloz@gmail.com; 3 Departamento de Cristalografía, Mineralogía y Química Agrícola, Universidad de Sevilla, c/Profesor García González 1, 41012 Seville, Spain; eboy@us.es; 4 Museo Nacional de Ciencias Naturales, Consejo Superior de Investigaciones Científicas (MNCN-CSIC). Serrano 115b., 28006 Madrid, Spain; humus@mncn.csic.es

**Keywords:** pyrogenic C, soil organic matter, humic acids, heavy metals, carbon sequestration, degraded soil

## Abstract

The application of biochar as an organic amendment in polluted soils can facilitate their recovery by reducing the availability of contaminants. In the present work, the effect of biochar application to acid soils contaminated by heavy metal spillage is studied to assess its effect on the quantity and composition of soil organic matter (SOM), with special attention given to soil humic acids (HAs). This effect is poorly known and of great importance, as HA is one of the most active components of SOM. The field experiment was carried out in 12 field plots of fluvisols, with moderate and high contamination by trace elements (called MAS and AS, respectively), that are located in the Guadiamar Green Corridor (SW Spain), which were amended with 8 Mg·ha^−1^ of olive pit biochar (OB) and rice husk biochar (RB). The results indicate that 22 months after biochar application, a noticeable increase in soil water holding capacity, total organic carbon content, and soil pH were observed. The amounts of oxidisable carbon (C) and extracted HAs in the soils were not altered due to biochar addition. Thermogravimetric analyses of HAs showed an increase in the abundance of the most thermostable OM fraction of the MAS (375–650 °C), whereas the HAs of AS were enriched in the intermediate fraction (200–375 °C). Spectroscopic and chromatographic analyses indicate that the addition of biochar did not alter the composition of the organic fraction of HAs, while Cu, Fe, and as were considerably accumulated at HAs.

## 1. Introduction

Soil pollution is one of the main environmental problems at a global scale. It affects 2.5 million sites in the European Union alone, with mineral oil and heavy metals the main contaminants contributing around 60% to soil contamination [1]. In fact, heavy metal-polluted soils represent 37% of the degraded sites in the European Union [2]. The high concentration of trace elements in soils is quite frequent in the Iberian Pyritic Belt, which is located in the southwest part of the Iberian Peninsula. These soils present serious functional problems, including difficulties for the development and establishment of microbial communities, as well as for the germination and growth of vegetation [3]. Recent estimates suggest that every year approximately 5 to 6 million ha of arable land are lost because of soil contamination, soil degradation, and marginalisation, which is an irreversible action. Given the environmental and economic dimensions of this land degradation process, soil contamination has been included as a priority in the EU environmental agenda.

The application of organic amendments offers a very good alternative to restore degraded soils [4,5]. This rehabilitation strategy usually improves soil’s physical and chemical properties, in addition to increasing the nutritional status, water infiltration rates, water holding capacity, and soil pH, which indirectly affect the sorption and complexation of metals in contaminated soils [6]. One of the most promising options is the transformation of biomass residues into biochar, the C-rich highly porous aromatic product resulting from the pyrolysis of biomass [7], for subsequent use as an organic soil amendment [8]. In fact, it has been reported that biochar has the ability to increase the pH of acidic soils with the consequent reduction in trace element bioavailability and immobilisation [9]. Its application to reclaim contaminated soils would allow the valorisation of organic waste and avoid ex situ treatments of contaminated soils, which are generally economically unfeasible [10]. Numerous recent studies show the agronomic benefits of applying biochar as an amendment in agricultural soils [11,12] and in soils contaminated with trace elements [9,13]. Biochar reduces the mobility of heavy metals [14] due to its high cation exchange capacity, alkaline pH, microporous structure, and surface functional groups. Numerous authors have suggested that biochar addition is a suitable strategy for the reclamation of heavy metal-polluted soils [15,16,17]. In fact, the application of biochar as soil ameliorant may change key physico-chemical properties of soils, such as soil pH, ion-exchange capacity, nutrient availability, organic carbon (OC), and dissolved organic matter, which results in effects on plant growth, the fate of inorganic and organic contaminants, and the microbial community structure [18]. Numerous studies have indicated that adding biochar increased heavy metal immobilization rates and decreased their bioavailability [19,20].

Investigations of trace element-polluted soils amended with biochar usually focus on the availability and amount of trace elements as well as on the biochar properties. However, the effects of biochar addition on SOM, which plays a key role in most of the biogeochemical reactions occurring in soils and is one of the determinants of soil quality and health [21], is in most cases neglected. Furthermore, because SOM responds relatively quickly to changes in both biotic and abiotic conditions, it is key to regulating and restoring the balance of environmental processes occurring in the soil, in what is defined as the “resilience” of the soil to recover from external variations [22]. Therefore, despite growing interest in biochar-based remediation, there is a knowledge gap concerning the effects of biochar on SOM composition when loading on soil environments. Campos et al. [4] showed that the application of biochar to soils contaminated with trace elements changed the microbial population 6 months after application, reducing the presence of extremophilic bacteria and increasing the presence of bacteria typical of non-contaminated soils in the Iberian Pyritic region. It has also been reported that soil environmental processes are affected by changes in biochar properties during ageing, such as changes in pore structure, sorption–desorption potential, and functional groups [23,24,25]. Biochar ageing may enhance its further degradation and cause the release of soluble organic compounds into the soil, affecting its humic fraction. Humic acid (HA) consists of a complex mixture of alkali-soluble complex functionalised organic molecules derived from the decomposition and oxidation of SOM. Some recent studies reported that biochar addition delivered humic matters to soil, which changed the micro-environment and activated As and Cu [26,27]. Nevertheless, the formation of humic substances has been considered as the major pathway for SOM stabilization and, thus, HAs are considered to possess a high resistance against biological decomposition [28].

Taking into account that the quality of SOM generally depends on the proportion and distribution of labile and recalcitrant forms, the higher quality and comparatively more resilient SOM being the one with a high degree of humification, high aromaticity, and complex molecular structure, the addition of biochar to the soil can alter the composition of SOM, and it is not known how it affects the composition of HAs and metal stabilisation. Alkaline conditions have been found to induce the preservation of the molecular structure of coal [29]. Nevertheless, the alterations that occur in the structure, composition, and stability of SOM when biochar is applied to soils contaminated with trace elements are quite unknown. Furthermore, little is known to date about the impact of biochar on soil quality under field conditions for temperate climates [30]. The importance of this issue is due to the fact that HAs are one of the most active components of SOM. Guo et al. [31] reported the utility of using HA fractions from the same soil source in order to understand SOM alterations.

This study proposes a comprehensive insight into SOM composition following biochar application in soils affected by a mining accident in 1998 that spilled about 4 hm^3^ of acidic waters and 2 hm^3^ of toxic sludge enriched with trace elements (especially, As, Cd, Cu, Pb, Tl, and Zn) into the Agrio and Guadiamar rivers basins (SW Spain), notably affecting soil properties, vegetation, and fauna of >4200 ha of agricultural and pasture lands. That spill was considered the largest mine spill in Europe to the date of the accident [32], and, despite the large-scale remediation activities performed, which included the removal of sludge and the topsoil, the subsequent application of organic and inorganic amendments, the afforestation with native plant vegetation, and the protection of the area by declaring it as a protected landscape called “Corredor Verde del Guadiamar”, the levels of some trace elements are still relatively high, and in some places the removal of the sludge was not completed and its toxic effects persist more than 20 years later [33].

Here, the combination of ^13^C NMR spectroscopy, Fourier transform infrared (FT-IR) spectroscopy, thermogravimetric analysis (TGA), differential scanning calorimetry (DSC), and analytical pyrolysis (Py-GC/MS) of HAs, in addition to the elemental composition and the analysis of soil physical properties, have been performed. Several previous studies have already successfully applied ^13^C NMR spectroscopy and Py-GC/MS techniques for the analytical characterisation of HAs in soils and sediments [34,35]. Due to the high complexity and heterogeneity of HAs, a combination of complementary analytical techniques is required to gain valuable information about their structure, composition, and functional features in biochar-amended and un-amended acid soils.

Assuming the above considerations, the major aims of this investigation were to: (1) explore the composition of HAs in soils contaminated with heavy metals affected by the Aznalcóllar spill in Seville Province in 1998; (2) discern possible changes in the composition of the HA fractions due to the biochar amendment; (3) establish the connection between biochar amendment, soil properties, and soil functionality for soils that are contaminated with trace elements.

## 2. Materials and Methods

### 2.1. Biochars

Biochar materials that were used for the present study were produced from rice husk and olive pit following the conditions described by De la Rosa et al. [36]. Briefly, the raw materials were subjected to a pyrolysis process up to 500 °C in an inert atmosphere (N_2_), with a residence time of 12 min in a Pyreka rotary pyrolysis reactor (Pyreg, Germany) fed by a screw. Hereafter, these biochars are referred to as: RB for rice husk biochar and OB for olive pit biochar.

### 2.2. Study Area and Field Experiment

**Figure 1:**
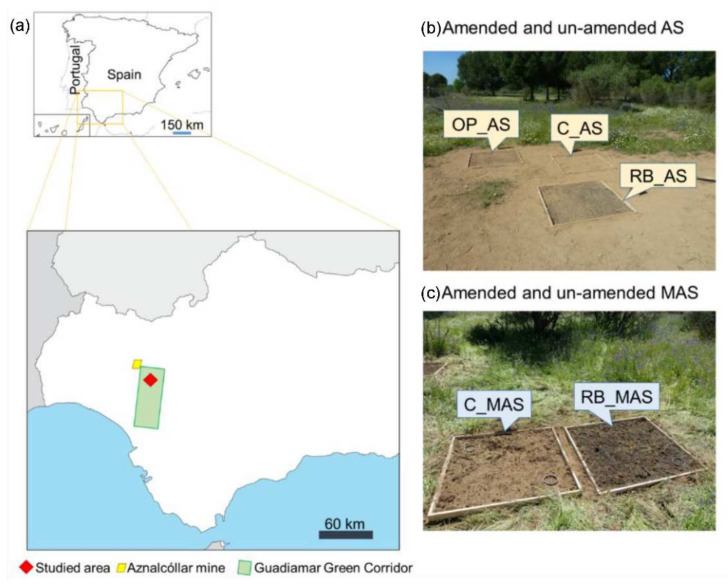
Location of the studied area (**a**) and views of the control and amended plots: acid contaminated soil (AS; **b**) and moderately acid contaminated soil (MAS; **c**).

The study area is located at “*Las Doblas*” site in the “Corredor Verde del Guadiamar” (37°23′7.152″ N, 6°13′43.175″ W; Sanlucar la Mayor, SW Spain; Figure 1), about 10 km South of the “Los Frailes” mining pond within the Iberian Pyritic Belt, which was breached in April 1998 and, consequently, dumped an enormous amount of acidic sludge that was contaminated with As, Pb, Cd, Cu, and Zn, among other trace elements [5,35]. The climate of the area is typical dry Mediterranean, with hot and extended summers, mild, rainy winters, a medium temperature of 17.7 °C, and an average annual rainfall of 400 mm [37]. The soils of this area are classified as fluvisols, IUSS Working Group WRB, 2015 [38], and are composed of 56–64% sand, 27–34% silt, and 8–10% clay (sandy loam soils). The field trial was carried out on plots with 2 different degrees of heavy metal contamination: acid contaminated sites (AS) and moderately acid contaminated sites (MAS). The whole study area was affected by the toxic spill, but the toxic acidic sludge was not effectively removed from the AS sites.

Vegetation of the area was studied previously via plant surveys and samplings [7,33,39]. Three species were deciduous (*Populus alba, Celtis australis*, and *Fraxinus angustifolia*) and evergreen (*Quercus ilex, Ceratonia siliqua*, and *Pinus pinea*), but the most dominant herbaceous and scrub species were *Poa annua, Lamarckia aurea, Chamaemelum mixtum*, *Cistus* sp., and *Cynodon dactylon*. Vegetation was practically absent at the AS sites. A more detailed description of the Aznalcóllar mine tailing accident and of the sites that were used for the present study can be found in Madejón et al. [33] and Campos et al. [4], respectively. In brief, 8 Mg·ha^−1^ of RB and OB were applied to 1 m × 1 m plots and subsequently mixed with topsoil (5 cm), leaving control plots without biochar (*n* = 3). About 22 months after the amendment, 3 soil samples were taken from each plot with a manual auger flat shovel, making cuts of 10 m × 10 m × 10 cm. All samples were transported in sealed bags and taken immediately to the laboratory, where they were dried at 40 °C for 48 h, sieved (<2 mm), homogenised, and stored in sealed bags at 4 °C.

### 2.3. Determination of Oxidisable Soil Organic Carbon

A colorimetric methodology adapted by FAO (2019) was followed to determine the oxidisable soil organic carbon (Ox-OC) content of each sample. This procedure is based on the wet oxidation of SOM proposed by Walkley and Black [40]. In brief, 0.5 g of dry soil sample and the necessary amount of glucose were weighed in order to make a standard line with a range of 0 to 8 mg C; 2 mL of 10% K_2_Cr_2_O_7_ solution was added and mixed with a stirring rod. Then, 5 mL H_2_SO_4_ was slowly added, stirred, and allowed to stand for 30 min. After adding 20 mL H_2_O and mixing, the solution was left to stand overnight, and then it was subsequently filtered, and the absorbance of the filtered extracts was measured at 600 nm. This determination was carried out in triplicate. Using the standard line, the amount of C present in the sample was determined, and the amount of Ox-OC was calculated in g·kg^−1^ using the exact weight of the initial dry soil sample.

### 2.4. Extraction of Soil Humic Acids (HAs)

The HA fractions were isolated from the dried soils following a procedure that was adapted from Schnitzer [41]. In brief, the soil samples (100 g) were shaken with a solution of 0.1 M Na_4_P_2_O_7_·10H_2_O + 0.1 M NaOH for 24 h under an N_2_ atmosphere. The alkali-soluble fraction (total humic extract) was separated by centrifugation at 6000 r.p.m. for 15 min and filtration, repeating the process five times until the extracts were almost transparent. The extracts were acidified to pH 1–1.5 by adding 6 M HCl, precipitating the HAs. The HA fraction was re-dissolved in 0.5 M NaOH and centrifuged at 6000 r.p.m for 15 min. The HA fractions were repeatedly washed and dialysed against milli-Q H_2_O using a Spectrapore membrane with a size exclusion limit of 6000–8000 Da, until the water was free of chlorides. Finally, the HA fractions were frozen and freeze-dried.

### 2.5. Physical Properties and Elemental Composition

Soil pH was measured with a Crison 40 pH meter (Crison Instruments SA, Barcelona, Spain) in a 1:10 (*w*/*v*) soil–distilled water mixture.

Total C and N contents of pure biochar, soils, and HAs were determined by dry combustion using an elemental analyser that is equipped with a thermal conductivity detector (Thermo Instruments, Bremen, Germany) at 1020 °C, which by catalysed combustion converts all C to CO_2_ and N to NO_2_.

Total soil moisture (TSM) (%) was determined by weighing 20 g of the samples before and after drying at 105 °C for 24 h.

Water holding capacity (WHC) was calculated in accordance with the work of Campos and De la Rosa [9] by weighing the soil sample before and after saturation with Milli-Q water and draining for 2 h. The WHC was calculated as the ratio between the mass of water retained by the sample at field capacity and the weight of dry sample, expressed as a percentage.

To determine total organic matter (loss on ignition at 750 °C), dry soil samples (105 °C) were placed in porcelain capsules containing 10 g of bulk soil, then kept in a muffle for 5 h at 750 °C.

Data of soil characteristics and composition are expressed as mean ± standard error (SE) of triplicate measurements.

### 2.6. Analytical Pyrolysis (Py-GC/MS) of Humic Acids

Analytical pyrolysis (Py-GC/MS) was conducted using a double-shot pyrolyser (model 2020i, Frontier Laboratories, Essen, Germany) operating in single shot mode, attached to a gas chromatograph (model 6890N, Agilent, Santa Clara, CA, USA). Using an inert atmosphere of He, approximately 4 mg of sample was introduced for one minute into a preheated (400 °C) micro-furnace. The evolved gases were then directly injected into the GC/MS for analysis. The GC was equipped with a low-polarity DB-5 (J&W Scientific, Palo Alto, CA, USA) fused silica capillary column (30 m length, 0.25 μm inner diameter, and 250 μm film thickness). The carrier gas was He at a controlled flow of 1 mL·min^−1^. A mass selective detector (model 5973MSD, Agilent, Santa Clara, CA, USA) was used with a 70 eV ionising energy.

Single ion monitoring (SIM) was applied to identify individual compounds for different homologous series. Furthermore, low-resolution mass spectrometry and the comparison of the mass spectra with stored data (NIST and Wiley libraries), and with previously published mass spectra, were used to identify the compounds.

### 2.7. Spectroscopic Analysis of Humic Acids

#### 2.7.1. Fourier Transform Infrared (FT-IR) Spectroscopy

The Fourier transform infrared (FT-IR) spectra were acquired with a Cary 630 ATR-FT-IR spectrometer with a diamond ATR sampling accessory (Agilent Technologies, Santa Clara, CA, USA). Spectra were obtained in the wavelength range of 4000–400 cm^−1^ by acquiring 50 scans at a resolution of 2 cm^−1^.

Spectral data were background corrected before each measurement. Spectra were smoothed and baseline corrected, and a digital filter was also applied for resolution enhancement in order to facilitate band identification in typical broadband spectra. The method used is based on subtracting from the raw spectrum a positive multiple of its 2nd derivative [42].

#### 2.7.2. Solid-State ^13^C NMR Cross-Polarisation Magic-Angle Spinning (CP-MAS) Spectroscopy

Dried and milled HAs were packed into 4 mm zirconia (ZrO_2_) rotors with Kel-F caps and analysed using a Bruker Avance III HD 400 MHz NMR Spectrometer (Bruker Corporation, Billerica, MA, USA) for obtaining the spectra. Information on acquisition parameters and analysis of the ^13^C NMR data are given in Table 1.

### 2.8. Thermal Analysis of Humic Acids

Thermogravimetric and differential scanning calorimetric (TG–DSC) analyses of HAs were conducted using a Discovery series SDT 650 simultaneous DSC/TGA instrument (TA Instruments Inc., New Castle, DE, USA) under the He flow rate of 50 mL·min^−1^. The HA samples (4 mg) were placed in alumina pans without cover and heated from 50 to 650 °C at a heating rate of 20 °C·min^−1^. TG, DSC curves, and mass loss were obtained via TRIOS software (TA Instruments, Delaware, DE, USA).

Thermogravimetric and differential scanning calorimetric (TG–DSC) analyses of HAs were conducted using a Discovery series SDT 650 simultaneous DSC/TGA instrument (TA Instruments Inc., New Castle, DE, USA) under the He flow rate of 50 mL·min^−1^. The HA samples (4 mg) were placed in alumina pans without cover and heated from 50 to 650 °C at a heating rate of 20 °C·min^−1^. TG, DSC curves, and mass loss were obtained via TRIOS software (TA Instruments Inc., New Castle, DE, USA).

### 2.9. Portable X-ray Fluorescence (pXRF) Analysis of Humic Acids

To obtain rapid information on the possible accumulation of trace elements in the HAs of these contaminated sites and to discern the effect of the application of biochar as a soil amendment on the abundance of trace elements, a portable X-ray fluorescence equipment was used. Thus, dried and finely ground HA samples were measured in an XRF analyser Niton XL3t 950s GOLDD+ XRF (Thermo Scientific Inc., Billerica, MA, USA), with its laboratory stand in desktop mode to increase precision and repeatability while performing analyses [44]. This technique allows a non-destructive analysis of the samples that can thus be used for other determinations. The analyser has two factory calibration modes, called mining mode and soils mode. The latter is based on Compton normalisation, which is commonly used for the detection of metallic elements that are present in soils at low concentrations (<1%). Each sample was scanned in triplicate, repositioning the sample container before each scan. In this mode, the analysis time for each scan was 60 s and readings were obtained for the following elements: Sr, Pb, As, Zn, Cu, Fe, and Ba. Furthermore, Ni and Cd were included in soil mode, although they were below the limit of detection (LOD) in all samples. The reference material SRM 2709 San Joaquin Soil [45], recommended for baseline trace element concentrations, produced by the National Institute of Standards and Technology (NIST, Spain), was used to assess the accuracy of the pXRF instrument. The measured value for each target analyte should be within ±20% relative difference (RD) of the true value in order to consider the verification check as acceptable [46].

### 2.10. Statistical Analysis

Data corresponding to the composition, basic properties, and concentrations of trace elements are shown as mean values ± standard error (SE) of three replicates. Biochar samples, soils taken at t_0_ and soils sampled at t_22_ have been considered independently. One-way analysis of variance (ANOVA) and Tukey’s Honestly Significant Difference (HSD) test were performed to study the effects of biochar application on soil properties and composition. The Kruskal—Wallis test followed by the Mann–Whitney U test were performed for non-normal variables. Statistical analyses were carried out using IBM SPSS Statistics 26.0 (SPSS, Chicago, IL, USA). A significant level *p* = 0.05 was used throughout the study.

## 3. Results and Discussion

### 3.1. Effects of Biochar Addition on Soil Composition, Properties, and Abundance of Organic Matter

The basic properties and composition of biochars, original soils, and amended and un-amended soil samples taken 22 months later are shown in Table 2.

Both biochars presented alkaline pH (>9), similar total moisture (3–4%), and low N content (1.6 and 5.1 g·kg^−1^ for OB and RB, respectively). High WHC was noted at RB, which has been related to its high Si composition and the formation of micro- and meso-pores during slow pyrolysis [47]. Analysis of the soils at time 0 revealed their acidity, pH was 3.6 and 4.8 for AS and MAS, respectively, with low OC and N content, as expected for degraded soils with little or no vegetation development and a high abundance of metals.

The amendment of the acid fluvisols for 2 years with biochar produced some changes in soil properties and composition, although most of them were not significant (*p* ≥ 0.05). Biochar application increased soil pH (up to 0.5 units when adding RB), OC content, moisture, and SOM content. These characteristics indicated that rice husk and olive pit biochar may improve soil quality and functionality. However, the contents of Ox-OC and HAs of the soils did not change 22 months after biochar application.

The AS soils contained less OC, OM, Ox-OC, and HAs than MAS soils. This result is a consequence of the impossibility to develop vegetation during the last 20 years since the mining tailings dam accident, and is a clear indication of the degradation degree of these acid sites.

### 3.2. Effects of Biochar Addition on Humic Acids

#### 3.2.1. Analytical Pyrolysis (Py-GC/MS)

**Figure 2:**
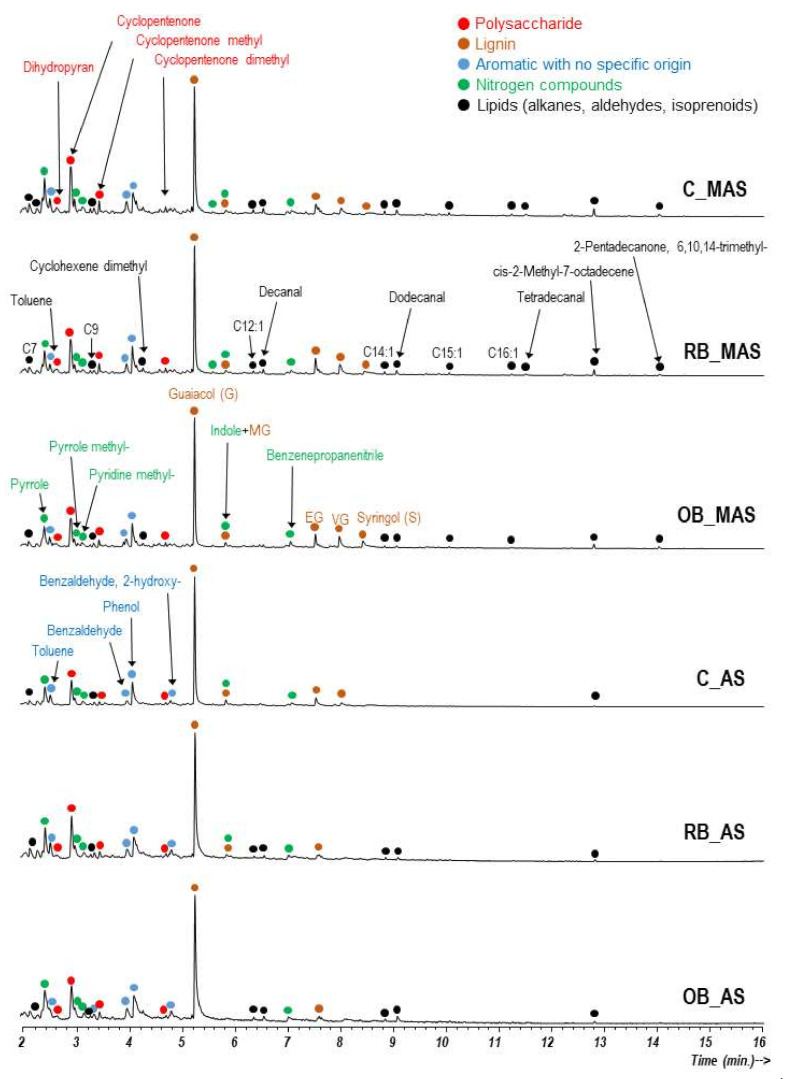
Py-GC/MS Total ion current chromatograms of the HA fraction isolated from moderately acid (MAS) and acid soils (AS) not amended (control) and amended with biochars produced from rice husk (RB) and olive pit (OB). Labels on the peaks indicate the origin of major pyrolysis compounds G: Guaiacol. MG: Methylguaiacol. EG: Ethylguaiacol. PG: Propyl-guaiacol. VG: Vinylguaiacol.

The total ion current chromatograms (TIC) obtained by GC/MS analysis of the HAs, generated by direct pyrolysis at 500 °C, are depicted in Figure 2.

The pyrolysates of HAs were dominated by methoxyphenols with guaiacyl (G) and syringyl (S) structure (guaiacol, syringol, and their -methyl, -ethyl, -vinyl, and -propenyl derivatives), that are typical pyrolysis compounds from lignin, dihydropyran, cyclopentanones (originated from polysaccharides, such as cellulose), and nitrogen-bearing structures that may derive from proteins and polypeptides. Other plant-derived compounds present are triplet peaks of *n*-alkanes, α-alkenes, and α,ω-alkanedienes (C_7_–C_16_), some aldehydes and *cis*-2-methyl-7-octadecene, probably from rests of epicuticular waxes, and the isoprenoid phytone (2-pentadecanone, 6,10,14-trimethyl-), with its origin in the chlorophyll side chains. A scarce number of aromatic compounds (toluene, phenol, and benzaldehydes) with uncertain origin, were also found that may be the end-products of polyphenols’ (lignin) degradation. All of these identified compounds are typical of humic substances [48]. Most of the N-containing compounds (including pyrroles and alkanenitriles) have a probable protein origin (microbial input). Therefore, the low proportions indicate little microbial activity.

All HAs that were extracted from the MAS and AS, amended or not with biochar, showed very similar pyro-chromatograms with no noticeable differences among them, except for the absence of some lipids on the HAs from the AS soils, which may correspond to the poor development of vegetation and microorganisms in these soils [47]. The absence of noticeable differences between the pyrograms of the HAs due to the application of biochar, compared to the HA extracted from the control soils, could suggest that the biochar has not been transformed into extractable colloidal substances that can be incorporated into the HA fraction, at least during the 2-year experiment. Furthermore, a specific search for possible char-derived compounds in the pyrolysates, e.g., bisphenols, naphthalene, or alkylnaphthalenes and other polycyclic aromatic hydrocarbons, was not successful. However, it should be noted that the pyrolysis temperature that was used for analytical pyrolysis (500 °C), is similar to that used to produce the biochars. Thus, it is not expected to observe highly recalcitrant organic compounds with this tool.

#### 3.2.2. Spectroscopic Analyses of Humic Acids

##### ^13^C Solid-State Nuclear Magnetic Resonance Spectroscopy of Humic Acids

**Figure 3:**
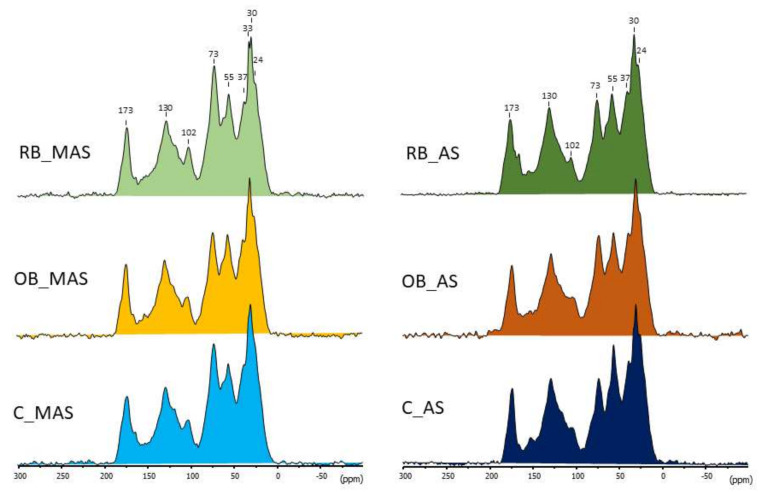
CP-MAS ^13^C NMR spectra of humic acids. C, OB, and RB labels refer to the samples taken from control (un-amended), olive pit biochar, and rice husk biochar-amended plots, respectively. MAS and AS indicate moderately acid and acid soils, respectively.

All of the HAs showed similar spectra and exhibited major peaks at 30 ppm (alkyl carbon), 55 ppm (methoxyl groups associated with lignin and lignin-like components), 73 ppm (*O*-alkyl carbon from to C-2, C-3, and C-5 carbons in polysaccharide structures), a prominent peak at 130 ppm (aromatic carbon), and a sharp peak centred at 173 ppm (assigned to carboxyl carbon; Figure 3).

The small peak at 24 ppm corresponds to short-chain, branched structures, or terminal methyl groups, whereas the peak at 33 ppm is typically assigned to alkyl-C in long chain methylene structures from lipid polymers or condensed wax material [49]. The relative intensity distribution of the different classes of C atoms, corresponding to the ^13^C NMR spectra of the six HAs shown in Figure 3, is given in the Supplementary Material (Table S1). The integration values of the different C types reflected no noticeable differences between the isolated HAs, irrespective of the degree of soil acidity or the application of biochar. A greater relative abundance of *O*-alkyl-C was observed in the HAs from MAS than in AS, 21–23% vs. 16–18%, respectively, which is probably due to some recent contribution of plant material at the MAS. No difference in the degree of aromaticity of the HAs was found.

##### Fourier Transform Infrared (FT-IR) Spectra of Humic Acids

**Figure 4:**
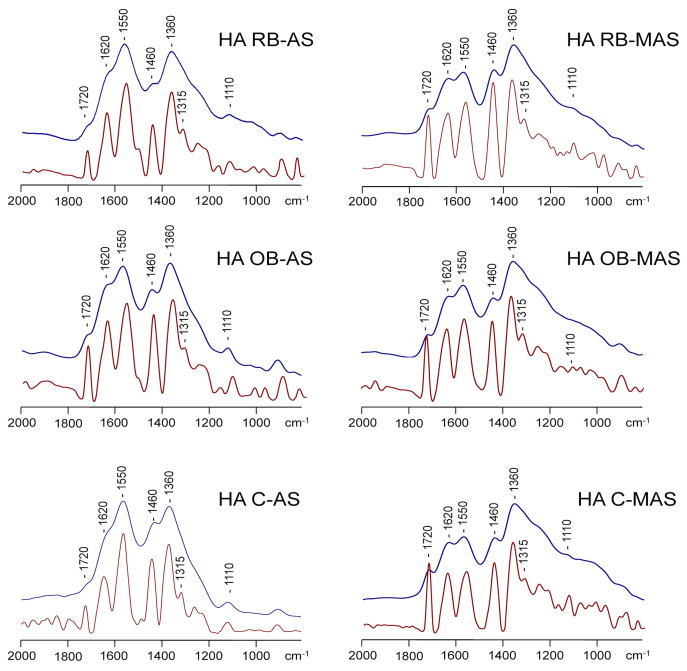
Fourier transform infrared (FT-IR) and resolution-enhanced IR spectra (below) of humic acids from biochar-amended and un-amended soils. Labels refer to the Section 2.

The FT-IR spectra of all the HAs were very similar, with a common pattern of bands (Figure 4). The intense peak at ca. 1550 cm^−1^ and the shoulder at 1620 cm^−1^ are assigned to C=C in aromatic rings, although the intensity of this region is produced in general by the overlapping of a series of bands of quinones and olefinic groups, as well as by accompanying substances such as water or minerals. Similarly, the signal centred at ca. 1360 cm^−1^ represents the C=C stretch of aromatic rings [50,51]. The scissoring mode of –CH_2_ group gives rise to the characteristic band near 1460 cm^−1^. The small peak centred at 1720 cm^−1^ suggests the presence of esters and/or acids. FT-IR spectra show a similar composition of the HAs that were observed using ^13^C NMR spectroscopy, without noticeable differences due to biochar addition to the plots, which is also indicative of the low solubility of biochar.

#### 3.2.3. Thermal Analysis of Humic Acids

Results corresponding to the thermogravimetric and differential scanning calorimetry (TG–DSC) analyses of HAs are shown in Table 3. The mass loss measured by TG can be associated with the decomposition of different sorts of OM [52,53]. The derivative of weight loss as a function of temperature permitted to identify the different types of OM, according to their thermal stability (see electronic annex; Figure S1). Consequently, the area under the TG curves was divided into four sections, representing different degrees of resistance to thermal oxidation: W1 moisture and very labile OM (50–125 °C): composed by water and volatile OM; W2 labile OM (125–200 °C): mainly polysaccharides; W3 intermediate OM (200–375 °C) composed of proteins and aliphatic compounds; W4 recalcitrant OM (375–650 °C), such as lignins, polyphenols, and condensed aromatic structures. The TGs of all the HAs were very similar. In general, differences were only observed when comparing the HAs extracted from MAS versus AS soil types, irrespective of whether or not biochar was added, or the type of biochar employed. In the case of the HAs obtained from the AS soils, it is observed that the most notable difference is the increase in the W3 fraction, corresponding to intermediate OM (from 28–31% to 36–39% of relative total weight loss). In contrast, HAs from MAS soils were enriched on the most recalcitrant OM (W4) with contributions ranging from 40 to 43% of the relative weight loss (Table 3).

Investigating DSC peaks information, concerning the different nature of the decomposed OM, could be obtained. Biological stability can be predicted from DSC data due to the similarity in the energy amount required for SOM decomposition and thermal oxidation [54,55].

All of the HAs showed two endothermic peaks at 90–99 °C and 119–124 °C, corresponding to the evaporation of water. The exothermic peaks that were present appeared with maxima at 300 °C, 403–420 °C, and 482–490 °C, the latter being the greatest for the HAs isolated from the MAS samples, which suggests the large thermal recalcitrance of the HAs from the moderately acid soils. This result could be due to the much higher microbial activity, as recently reported by Campos et al. [25] in the MAS, in comparison with the AS sites.

#### 3.2.4. Metal Concentrations of Soils and Humic Acids

Table 4 shows the concentrations of As, Ba, Cu, Fe, Pb, Sr, and Zn (µg·g^−1^) on a dry weight basis in bulk soils AS and MAS, and the corresponding HAs. The total contents of those trace elements, recorded for soils AS and MAS, show a high concentration of the seven elements that were analysed. The measured values are in good agreement with the values reported previously by Campos and De la Rosa [9] in the same area with determinations carried out by acid digestion and measured by ICP-OES. It is noteworthy that the high Fe content of both soils is predictably related to the dumping of pyrite mining waste that contaminated them. There is also concern about the abundance of Pb due to its danger, considering the acidity of the AS sites, which increases the risk of bioavailability as well as the high concentrations of As. These concentration values of trace elements prevent these soils from being suitable for agricultural use, according to the regional regulations of the Andalusian Government (As: 36 µg·g^−1^, Pb: 275 µg·g^−1^), and are high enough to imply a mandatory level of investigation [56].

Considering that the process of extracting HAs requires several sequential steps of solubilisation of SOM at very alkaline pH (with NaOH), followed by precipitation in acidic media (HCl), subsequent washing, re-solubilisation, re-precipitation, washing, and dialysing, one should not expect to measure high concentrations of metals in the HAs.

Nevertheless, it is observed that HAs, in relation to the soil concentrations, accumulated greater concentrations of Cu and Fe, similar concentrations of As, and lower concentrations of Ba. Previous authors also reported relative enrichment factors of trace elements at HAs up to 30×, thus corroborating: (i) the importance of HAs as a geochemical carrier for metal ions, as a driver on the mobility and distribution of heavy metals in soils, and (ii) the strong affinity of Cu, Fe, and As for HAs [28,57]. The data on Cu accumulation in HAs are particularly striking, as they are even significantly higher than the values measured in the spilled pyritic toxic sludge (mean Cu content of 1552 mg·kg^−1^ according to Madejón et al. [33].

## 4. Conclusions

This study demonstrates that the combination of elemental, chromatographic, and spectrospic analyses provides valuable information concerning the impact of biochar on the composition of HAs and the selective metal–HA interactions. Spectroscopic and analytical pyrolysis analyses of soil HAs showed no noticeable differences between the isolated HAs, irrespective of the degree of soil acidity or the application of biochar, except for a slightly greater abundance of *O*-alkyl-C in the HAs from MAS, rather than from AS. Thermogravimetry results showed a great similarity of the thermal stability of all the HAs, without variations with the application of biochar as an amendment, and the only remarkable difference was the greater abundance of recalcitrant OM in the HAs of the MAS, in comparison with that of the AS. The application of 8 Mg·ha^−1^ of biochar produced from agricultural residues on acid fluvisols polluted with trace elements increased pH, WHC, and OC content of the amended soils but, contrary to what had been previously published, did not affect the abundance or composition of the HAs. The study of trace elements of the extracted HAs revealed that they sequester considerable amounts of Cu and Fe, and to a lesser extent As, with concentrations up to more than 18 times greater than those of the corresponding bulk soil in the case of Cu, indicating the importance of HAs with respect to the transport and fixation of trace elements in soils. This study demonstrates the importance of HAs in the stabilisation of heavy metals in soils and the lack of changes in HA composition 2 years after biochar application. The stability of the organic fraction of biochar-amended soils, added to the increase in the amount of stable carbon, imply a high resilience of the biochars used as soil amendments for the recovery of acid soils contaminated with trace elements under typical Mediterranean climate conditions. This result is of relevance to the implementation of the application of biochar from local agro-waste in policies for the management of land contaminated with heavy metals and to establish adequate strategies for in situ remediation of the effects of these persistent pollutants in soils.

## Figures and Tables

**Table 1:** ^13^C NMR spectra acquisition parameters and chemical shift assignment of the peaks.

Spinning Speed:	14 kHz
Contact time:	1 ms
Number of scans per sample:	4000 to 6000
^13^C calibration:	Tetramethylsilane (0 ppm) and glycine (COOH at 176.08 ppm)
Signal (ppm)	Assignment [43]
0–45	Alkyl-C
45–60	*N*-alkyl-C/methoxyl C
60–90	*O*-alkyl (carbohydrates, alcohols)
90–160	Sp^2^-hybridized C; Aryl C + intensity of the spinning side bands
160–185	Carboxyl and amide-C
185–220	Carbonyl C (aldehyde and ketone C)
Integration software:	MestReNova 10 (Mestrelab Research, Santiago de Compostela, Spain)

**Table 2:** Basic properties and composition of biochars and bulk soils (t_0_ and t_22_).

Samples		Code	Properties			Composition				
			pH	WHC (%)	Total Moisture (105 °C; %)	OC (g·kg^−1^)	N (g·kg^−1^)	OM (750 °C, %)	Ox-OC (g·kg^−1^)	Humic Acids (%)
Biochars	Rice husk biochar	RB	10.1 ± 0.3 ^a^	540 ± 77 ^a^	3.3 ± 0.4	536.6 ±1.4 ^a^	5.1 ± 2.4 ^a^			
	Olive pit biochar	OB	9.3 ± 0.2 ^b^	66 ± 6 ^b^	4.1 ± 0.2	927.1 ±1.6 ^b^	1.6 ± 0.9 ^b^			
Soils before amendment (t_0_)	Moderately acid soil	MAS	4.8 ± 0.1 ^a^	43 ± 4 ^a^	8.3 ± 0.4	13.0 ± 1.0 ^a^	2.0 ± 0.7	5.8 ± 0.6		
	Acid soil	AS	3.6 ± 0.1 ^b^	33 ± 2 ^b^	7.2 ± 0.6	9.2 ± 0.9 ^b^	0.9 ± 0.4	5.0 ± 0.5		
Soils from field experiment (t_22_)	Control_moderately acid soil	C_MAS	5.5 ± 0.1 ^c^	47 ± 6	10.3 ± 0.5 ^b^	16.2 ± 1.3 ^b^	2.0 ± 0.1	6.1 ± 0.2 ^b^	10 ± 1	1.3
	Rice husk biochar_ moderately acid soil	RB_MAS	6.0 ± 0.2 ^d^	52 ± 2	13.8 ± 0.3 ^c^	20.3 ± 2.2 ^b,c^	1.9 ± 0.2	8.4 ± 0.3 ^c^	11 ± 1	1.2
	Olive pit biochar_moderately acid soil	OB_MAS	5.8 ± 0.2 ^c,d^	58 ± 5	13.3 ± 0.5 ^c^	22.4 ± 2.9 ^c^	2.2 ± 0.5	7.5 ± 0.9 ^c^	13 ± 2	1.1
	Control_acid soil	C_AS	3.5 ± 0.2 ^a^	34 ± 3	7.1 ± 0.5 ^a^	11.5 ± 0.6 ^a^	1.6 ± 0.4	5.0 ± 0.3 ^a^	7 ± 2	0.8
	Rice husk biochar_acid soil	RB_AS	4.0 ± 0.1 ^b^	41 ± 7	10.2 ± 0.1 ^b^	16.7 ± 1.5 ^b^	2.0 ± 0.3	7.0 ± 0.4 ^c^	7 ± 1	0.8
	Olive pit biochar_acid soil	OB_AS	3.8 ±0.2 ^a,b^	31 ± 5	10.8 ± 0.5 ^b^	18.4 ± 2.0 ^b^	2.1 ± 0.3	7.1 ± 0.5 ^c^	8 ± 1	0.8

t_0_: Day 0 of the experiment (before the amendment). t_22_: 22 months after the amendment. WHC: water holding capacity. OC: total organic carbon content. OM: total organic matter content by loss on ignition. Ox-OC: oxidisable organic carbon content by wet oxidation. Different letters within a column (^a,b,c,d^), for the same time (t_0_ or t_22_) and type of sample (biochar or soil), indicate significant differences between treatments (*p* < 0.05). No letter indicate no significant differences.

**Table 3:** Comparative thermogravimetry (TG) and differential scanning calorimetry (DSC) parameters in HA samples summarizing: Total weight loss for the temperature interval 50–650 °C (% ± 1%), weight losses and relative weight losses for the temperature intervals, 50–125 °C (W1), 125–200 °C (W2), 200–375 °C (W3), 375–650 °C (W4), and temperature of the exothermic peaks.

Code		MAS			AS		
		C_MAS	RB_MAS	OB_MAS	C_AS	RB_AS	OB_AS
TG	Temperature Interval						
Total Weight Loss (%)	50–650 °C	46.0	42.4	42.8	43.5	44.8	45.5
Moisture and very labile OM-W1	50–125 °C	9.0	7.8	7.4	7.0	8.1	8.1
Labile OM-W2	125–200 °C	5.5	4.4	3.8	6.7	5.4	6.6
Intermediate OM-W3	200–375 °C	13.1	13.1	13.1	16.9	16.3	17.1
Recalcitrant OM-W4	375–650 °C	18.4	17.1	18.6	12.9	15.1	13.7
Relative Weight Loss (%)							
W1	50–125 °C	20	18	17	16	18	18
W2	125–200 °C	12	10	9	15	12	14
W3	200–375 °C	28	31	30	39	36	38
W4	375–650 °C	40	40	43	30	34	30
DSC Peaks (°C)	DSC endothermic peaks-W1	99, 121	98, 122	75, 123	90, 121	81, 122	119
	DSC exothermic peak-W2	n.d.	n.d.	n.d.	n.d.	n.d.	n.d.
	DSC exothermic peak-W3	296	295	299	295	301	298
	DSC exothermic peak-W4	415, 482	419, 489	411, 497	407	405, 490	403, 489

C: control (un-amended). OB: olive pit biochar-amended plots. RB: rice husk biochar-amended plots. MAS: moderately acid soil. AS: Acid soil. n.d.: Not detected.

**Table 4:** Concentrations of As, Ba, Cu, Fe, Pb, Sr, and Zn (µg·g^−1^) in bulk soils (t_0_) and humic acids in the studied area.

		As	Ba	Cu	Fe	Pb	Sr	Zn
Bulk soils (t_0_)	MAS	93 ± 5 ^a^	160 ± 32	215 ± 10 ^a^	44,985 ± 1220 ^a^	154 ± 7 ^a^	115.5 ± 1.6	275 ± 7 ^b^
	AS	254 ± 3 ^b^	187 ± 6	240 ± 6 ^b^	59,661 ± 1715 ^b^	401 ± 11 ^b^	117.2 ± 2.2	189 ± 6 ^a^
Humic acids (t_22_)	C_MAS	96 ± 6 ^b^	96 ± 2	2750 ± 46 ^a^	1847 ± 62 ^d^	17 ± 4	3.9 ± 1.2	75 ± 10
	RH_MAS	65 ± 8 ^a^	95 ± 3	2924 ± 46 ^b^	2132 ± 64 ^e^	21 ± 4	3.8 ± 1.2	56 ± 9
	OP_MAS	86 ± 5 ^b^	97 ± 2	3810 ± 50 ^d^	2408 ± 65 ^f^	22 ± 5	3.5 ± 1.1	61 ± 10
	C_AS	300 ± 9 ^d^	92 ± 4	3301 ± 50 ^c^	1005 ± 48 ^b^	<LOD	2.8 ± 1.2	34 ± 9
	RH_AS	218 ± 7 ^c^	93 ± 3	3297 ± 47 ^c^	757 ± 40 ^a^	<LOD	3.1 ± 1.1	53 ± 9
	OP_AS	205 ± 8 ^c^	91 ± 3	4307 ± 56 ^e^	1174 ± 50 ^c^	<LOD	3.3 ± 1.2	83 ± 12

t_0_: Day 0 of the experiment (before the amendment). t_22_: 22 months after the amendment. C: control (un-amended). OB: olive pit biochar-amended plots. RB: rice husk biochar-amended plots. MAS: moderately acid soil. AS: Acid soil. <LOD: below limit of detection. Different letters (^a,b,c,d,e,f^) within a column for the same time (t_0_ or t_22_) indicate significant differences between treatments (*p* < 0.05). No letter indicate no significant differences.

## Data Availability

The raw data are not publicly available but can be obtained via a reasoned request to the corresponding author.

## Supplementary Materials

The following supporting information can be downloaded at: https://www.mdpi.com/article/10.3390/ijerph19042140/s1, Figure S1: Derivative of weight loss vs. temperature from 50 to 650 °C of humic acids at a heating rate of 20 °C min−1. Table S1: Assessment (%) of the different C types of humic acids as seen by ^13^C NMR integration regions.
