# Supplementary material for: Impact of Biochar Amendment on Soil Properties and Organic Matter Composition in Trace Element-Contaminated Soil
Source: Int J Environ Res Public Health. 2022 Feb 14;19(4):2140. doi: 10.3390/ijerph19042140 (PMC8871668; doi:10.3390/ijerph19042140)
Supplement: Supplementary file 1 [file ijerph-19-02140-s001.zip › ijerph-1565891-supplementary.pdf]

Impact of biochar amendment on soil properties and organic matter composition in trace element-contaminated soil

SUPPLEMENTARY MATERIAL:

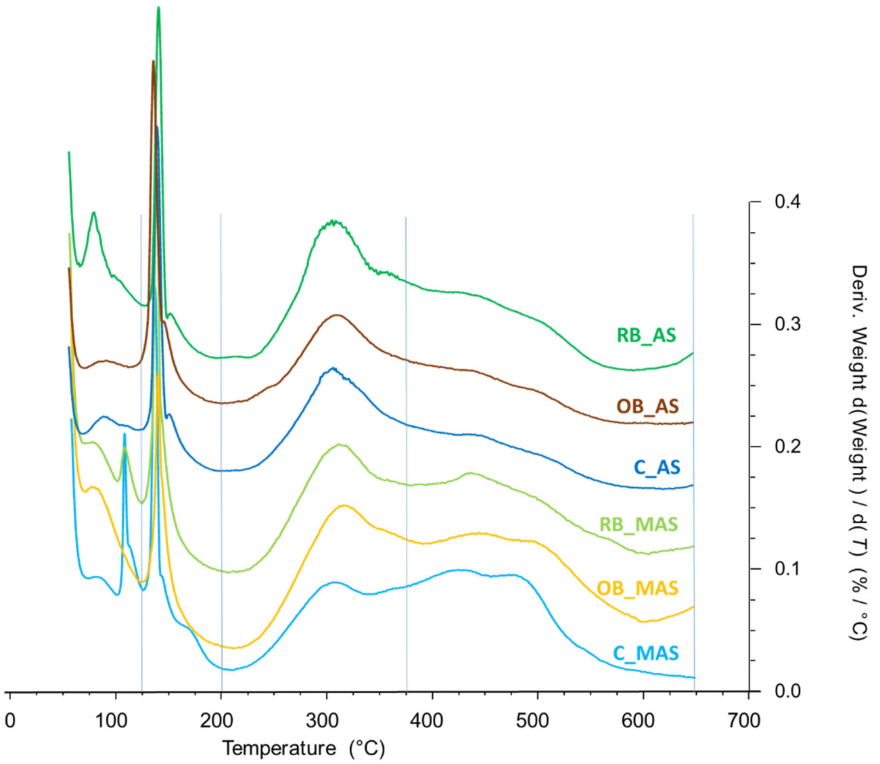

Figure S1. Derivative of weight loss vs. temperature from 50 to 650 °C of humic acids at a heating rate of 20 °C min^{-1}

Supplementary Table:

Table S1. Assessment (%) of the different C types of humic acids as seen by ^{13}C NMR integration regions.

Sample	245-185	185-160	160-110	110-90	90-60	60-45	45-0
**RB_MAS**	0	8	20	6	23	12	31
**OB_MAS**	0	9	21	6	20	12	32
**C_MAS**	1	9	20	6	21	12	31
**RB_AS**	1	11	22	5	17	12	33
**OB_AS**	1	9	22	6	18	12	32
**C_AS**	0	9	23	5	16	13	34

C region values are given in ppm.
